# Site-specific agronomic information and technology adoption: A field experiment from Ethiopia^☆^

**DOI:** 10.1016/j.jdeveco.2021.102788

**Published:** 2022-05

**Authors:** Hailemariam Ayalew, Jordan Chamberlin, Carol Newman

**Affiliations:** a Oxford Department of International Development, University of Oxford, UK; b International Maize and Wheat Improvement Center (CIMMYT), Nairobi, Kenya; c Department of Economics and Trinity Impact Evaluation Unit (TIME), Trinity College Dublin, Ireland

**Keywords:** Advisory services, Smallholder agriculture, Agricultural extension, Fertilizer, Agriculture, Digital agriculture

## Abstract

Smallholder farmers in Africa typically only have access to blanket fertilizer recommendations which are defined over very broad areas and may not be optimal for local production conditions. The response to such recommendations has generally been poor. Using a randomized control trial in Ethiopia, we explore whether targeted extension advice leads farmers to align fertilizer usage to the recommended levels and whether this impacts productivity. We also consider whether coupling the targeted information with agricultural insurance encourages fertilizer investment. Results show that targeted recommendations closed the gap between the amount of fertilizer used and the recommended amounts and this in turn increased productivity and profits. We found no differential effect of the targeted recommendation when coupled with agricultural insurance, suggesting that the risk of crop failure is not a binding constraint to fertilizer adoption in this context, or that farmers do not consider agricultural insurance a useful risk-mitigating mechanism.

## Highlights

• Most smallholders only have access to blanket fertilizer recommendations which may not be optimal for localized conditions.
• Site-specific fertilizer recommendations can reduce the gap between the amount of fertilizer used and recommended amounts.
• Adoption of targeted fertilizer recommendations increases productivity and profits.
• Coupling targeted fertilizer recommendations with agricultural insurance had no additional effect in our study.
• Results suggest that poorly defined extension information may constitute an important constraint to technology adoption.

## 1 Introduction

Agricultural productivity growth is one of the main components of the structural transformation process through which developing countries modernize and experience productivity and welfare improvements (Timmer, 1988; Diao et al., 2010; Christiaensen et al., 2011).^1^ Broad-based agricultural growth, by allowing greater participation by the poor in the growth process, has better poverty-reducing characteristics than growth which is concentrated in the commercial farm sector (Diao et al., 2010). Key drivers of such growth include improved crop varieties, inorganic and organic fertilizers, and other complementary agronomic management practices.

Technology adoption by smallholder farmers in developing countries has, however, remained persistently low over recent decades, even where such technologies are ostensibly profitable for farmers to use (Duflo et al., 2009, 2011; Suri, 2011; Emerick et al., 2016; Sheahan and Barrett, 2017). According to the World Bank (2007), fertilizer use in Africa is much lower than the rest of the world. Various reasons have been proposed for explaining such low adoption rates, including: technologies that are ill-suited to local conditions (Emerick et al., 2016); lack of information and difficulties in learning (Ashraf et al., 2009; Hanna et al., 2014); absence of formal insurance (Karlan et al., 2014); liquidity constraints (Croppenstedt et al., 2003; Gine and Klonner, 2008; Matsumoto and Yamano, 2011; Zerfu and Larson, 2010); high transaction costs due to poor infrastructure (Suri, 2011); and procrastination and time-inconsistent preferences (Duflo et al., 2011).

In this paper, we consider two specific constraints to technology adoption^2^: 1) the suitability of the technologies to local conditions; and 2) the downside risk associated with investing in a new technology due to potential crop failure. The context for our study is Ethiopia and the technology is a targeted site-specific fertilizer blend recommendation. We examine the impact of providing site-specific agronomic information (SSI) to smallholder farmers on fertilizer usage, farm productivity, profits from maize production, and household welfare using a two-level cluster randomized control trial. We conducted the field experiment in core maize producing zones of Ethiopia; namely West Gojjam, Jimma, East Showa, West Showa, and East Wellega zones. We randomized the provision of SSI and access to crop insurance across 130 1 km × 1 km grid-cells which cover, on average, 6 households in each cell. The agronomic information provided to treated households consisted of a site-specific fertilizer blend recommendation, the expected yield outcome at the recommended rate, and the optimal timing of fertilizer application. In a second treatment arm we couple this information with a free insurance product that protects farmers against crop failure due to weather-related events.

Soil degradation and nutrient depletion have been serious threats to agricultural productivity and food security in Ethiopia.^3^ Over the years, soil fertility has also declined due to the increase in population size and decline in plot size. Nitrogen (N) and phosphorus (P) are the nutrients that are most lacking (Murphy, 1968; Kebede and Yamoah, 2009; Spielman et al., 2010). In 2007, the Ministry of Agriculture and Natural Resources (MoANR) and Agricultural Research Centers together developed regional or blanket fertilizer recommendations (fertilizer types and application rates for different crops). These recommendations inform farmers how much fertilizer in kilograms they should apply per hectare without performing any soil test and regardless of agro-ecological zones. Since nutrient management requirements of farms vary across crop type, soil type and other agro-ecological characteristics, these blanket fertilizer recommendations may not be suitable for all farmers. The adoption of fertilizer remains low and the average application rates by those who do use fertilizer are generally lower than recommended rates.^4^

Our main finding is that well-targeted fertilizer recommendations induce greater fertilizer investments and improve the productivity of maize production. More specifically, we find that farmers who received SSI significantly closed the absolute gap between the actual total average macronutrient use and the recommended value by 18.6 kilograms per hectare (kg/ha).^5^ We find a similar effect for households who received the SSI coupled with the crop insurance cover but do not find any difference between the two treatment arms, on average. This suggests that crop insurance cover does not matter for the adoption of the recommended level of fertilizer use within our study context. We also find a noticeable increase in maize production for plots cultivated by treated farmers, both for those who received the information only and those who received the information with the crop insurance cover. The effect is large, amounting to a 0.5 metric tons per hectare (MT/ha) increase in average maize yield for plots cultivated by treatment farmers. Compared to the control group, the introduction of site-specific agronomic information improves maize productivity by around 15 percent. We also find that this translates into higher profits from maize production, although we do not find any evidence that this leads to improvements in household consumption.

Our study adds to the existing literature in three main ways. First, despite extensive literature on the factors that determine the adoption of fertilizer use, to the best of our knowledge there is no empirical study that assesses whether the relevance of the agronomic information for local conditions is itself a constraint to technology adoption. To the extent that African smallholder farmers have access to fertilizer recommendations, these are typically blanket recommendations, where the application rate and type of fertilizer recommended for a particular crop does not vary over a large (often national) area. Since the nutrient management requirements of farms vary across crop type, soil type and agro-ecological zone, blanket fertilizer recommendations may not be well-suited to all farms and may therefore give sub-optimal results. This in turn may affect farmers' fertilizer adoption rates in the future. This study tests whether fertilizer recommendations are more likely to be adopted when tailored to particular locations, and accompanied by detailed information on application methods, timing, and expected yields.

Second, in addition to the information channel, we examine the interactive effects of eliminating downside risk by providing insurance cover for crop failure with the site-specific agronomic information. Emerick et al. (2016) focus on the elimination of downside risk by providing a flood-tolerant rice variety to farmers. They find that the behavior of farmers changes once the downside risk is removed; they are willing to invest in more fertilizer and other inputs. Our study builds on this finding by providing additional evidence in a different context on the extent to which risk is a constraint to technology adoption when optimal information is available. That is, the elimination of downside risk may or may not be an important factor once farmers have the right information.

Third, our study is related to the recent literature exploring the demand for agricultural insurance and in particular, the reasons why the take-up of agricultural insurance is so low in developing countries. A number of studies have identified poor understanding of insurance mechanisms as a key factor in the low demand for insurance by smallholder farmers (Njue et al., 2018; Sibiko and Qaim, 2017). Other studies suggest that a lack of trust in the system and high premiums contribute to the low level of demand (Cai et al., 2009; Ceballos et al., 2019; Fonta et al., 2018). Our results suggest that even when agricultural insurance is given for free and its benefits explained clearly to farmers it does not seem to affect production decisions.

The rest of this paper is organized as follow. Section 2 presents a conceptual framework for understanding the relationship between information, insurance and technology adoption. In section 3, we explain the experimental design while the data and empirical approach are described in section 4. Section 5 presents the results and section 6 concludes.

## 2 Conceptual framework

In this section, we develop a simple theoretical framework that maps the relationship between site-specific agronomic information, insurance, and technology adoption along the lines of Magruder (2018). For simplicity, we assume a two-period model where farmers decide their investment and savings decisions in the first period and realize output in the second period. In the first period, the farmer decides to allocate income (y) either to purchase an input (x) or invest in a savings asset (a) which has a return (R) in the second period. The amount of input to be purchased during the planting period depends both on the state of the world (s∈S) that will be realized in the second period after the crop has been planted and the production function (t∈T). Assume $\theta_{s}$ is the probability that the state of the world, s, occurs and $\theta_{t}$ is the probability that any technological realization, t, occurs. Incomplete information implies that farmers are uncertain about the state of the world and how their input choices will perform in each state. The state-specific production function is given by $f_{s,t}\left(x\;\right)$, where $f_{s,t}^{\prime }\left(x\right)>0$, and $f_{s,t}^{\prime \prime }\left(x\right)<0,\;\forall s,t$. In period one, the farmer expects to produce ${\text{Ε}}_{s,t}\left[f_{s,t}\left(x\right)\right]$.

The farmer maximizes utility as follows:

(1) $$Max\;U=u\left(c^{0}\right)+\delta \underset{s,t\varepsilon S\times T}{\sum }\theta_{s}\theta_{t}u\left(c_{s,t}^{1}\right)$$

Subject to

(2) $$c^{0}=y-x-a$$

(3) $$c_{s,t}^{1}=f_{s,t}\left(x\;\right)+Ra$$

(4) $$x\geq 0,\;a\geq 0,\;a\leq \bar{a}$$

Where $c^{0}$ and $c_{s,t}^{1}$ are the levels of household consumption in the first and second period respectively, and δ is the discount factor.

The first order conditions with respect to x and a are:

(5) $$u^{\prime }(c^{0})=\delta \sum_{s,t\varepsilon S\times T}\theta_{s}\theta_{t}u^{\prime }\left(c_{s,t}^{1}\right)f_{s,t}^{\prime }\left(x\right)$$

(6) $$u^{\prime }\left(c^{0}\right)=\delta R\text{Ε}\left[u^{\prime }\left(c_{s,t}^{1}\right)\right]+\lambda_{a}$$

The two first order conditions imply:

(7) $$\delta R+\frac{\lambda_{a}}{\text{Ε}\left[u^{\prime }\left(c_{s,t}^{1}\right)\right]}=\delta \left(\text{Ε}\left[f_{s,t}^{\prime }\left(x\right)\right]+\frac{cov\left[f_{s,t}^{\prime }\left(x\right),u^{\prime }\left(c_{s,t}^{1}\right)\right]}{\text{Ε}\left[u^{\prime }\left(c_{s,t}^{1}\right)\right]}\right)$$

Equation (7) shows an optimal choice of input and savings that equates expected marginal benefits from the next period and marginal costs of forgone consumption in the current period. In this setup, farmers do not know which *t* of *T* is being realized and so have some uncertainty about the exact nature of the production function.

First, let us consider a scenario which assumes farmers have full information on t but still do not know the exact state of the world, s, that will be realized. That is, there is no uncertainty around what technology *t* is realized but they remain uncertain about the state of the world, *s*. This implies that $f_{s,t}^{\prime }\left(x\right)=f_{s}^{\prime }\left(x\right)$ and $c_{s,t}^{1}=c_{s}^{1}$. Under this scenario, the first order conditions can be re-written as:

(8) $$\delta R+\frac{\lambda_{a}}{\text{Ε}\left[u^{\prime }\left(c_{s}^{1}\right)\right]}=\delta \left(\text{Ε}\left[f_{s}^{\prime }\left(x\;\right)\right]+\frac{cov\left[f_{s}^{\prime }\left(x\;\right),u^{\prime }\left(c_{s}^{1}\right)\right]}{\text{Ε}\left[u^{\prime }\left(c_{s}^{1}\right)\right]}\right)$$

In both scenarios, the difference between the actual and expected output is higher if there is uncertainty about the state of the world, but in scenario 1, i.e. equation (7), there is also uncertainty about how input choices will perform in each state.^6^ That is, ${f_{s,t}}^{\prime }\left(x\;\right)-\text{Ε}({f_{s,t}}^{\prime }\left(x\;\right))$ $\geq \;{f_{s}}^{\prime }\left(x\;\right)-\text{Ε}({f_{s}}^{\prime }\left(x\;\right))$ and $u^{\prime }\left(c_{s,t}^{1}\right)-\text{Ε}(u^{\prime }\left(c_{s,t}^{1}\right))$ $\geq \;u^{\prime }\left(c_{s}^{1}\right)-\text{Ε}(u^{\prime }\left(c_{s}^{1}\right))$. As a result, $cov\left[{f_{s}}^{\prime }\left(x\;\right),u^{\prime }\left(c_{s}^{1}\right)\right]\;>cov\left[{f_{s,t}}^{\prime }\left(x\;\right),u^{\prime }\left(c_{s,t}^{1}\right)\right]$ and both terms are negative. This implies that, by reducing uncertainty on how input choices will perform in each state, improved information increases the amount of input used during the planting period.

Second, let us assume another scenario where there is full information and perfect insurance cover to mitigate downside risk. In this case, ${f_{s,t}}^{\prime }\left(x\;\right)={f_{s}}^{\prime }\left(x\;\right)$ and $c_{s,t}^{1}=c_{s}^{1}=c_{s}^{1I}\forall s$, where *I* indicates perfect insurance cover. Equation (7) can be rewritten as:

(9) $$\delta R+\frac{\lambda_{a}}{u^{\prime }\left(c_{s}^{1I}\right)}=\delta \text{Ε}\left[{f_{s}}^{\prime }\left(x\;\right)\right]$$

If we compare equation (7) and equation (9), we can see that since $cov\left[{f_{s}}^{\prime }\left(x\;\right),u^{\prime }\left(c_{s}^{1}\right)\right]<0$, the existence of (uninsured) risk in equation (7) reduces the input amounts used.

To sum up, incomplete information increases the uncertainty about the state of the world and how input choices will perform in each state. As a result, farmers will be reluctant to invest. Better information leads to lower outcome uncertainty and hence higher investments, *ceteris paribus*. The design of our experiment allows us to link better information (site-specific agronomic information on the amount of fertilizer use, timing of fertilizer application and expected yield for the recommended amount) and perfect insurance (free crop insurance in the event of a weather-related shock) to farmers' investment decisions (adoption of fertilizer recommendation) and productivity.

## 3 Sampling and experimental design

**Fig. 1:**
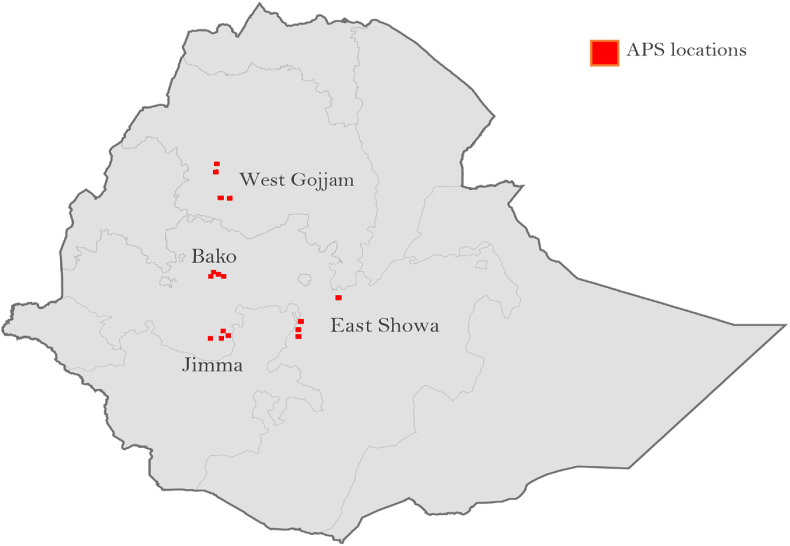
Map of the study area.

Source: 2017 Agronomic Panel Survey (APS) data

Our sample was drawn from the main maize growing areas of Ethiopia (Jimma, Bako, West Gojjam, and East Shewa). We randomly generated four 10 × 10 kilometer (km) sampling grids within each of these zones and subdivided each grid into 100 one km^2^ grid cells (Fig. 1). Within each grid, eight grid cells were chosen randomly. If a randomly selected grid cell could not be included (either because it was physically inaccessible or if there was no maize production taking place at or near that point), then a replacement location was drawn from the same 10 km × 10 km grid.

Within each of the randomly selected grid cells, six farmers were identified, using the following protocol. First, we identified the farm household closest to the selected point. For example, if the point fell within a field, we identified the farmer who owned the field. If this farmer grew maize in the current year, then this farmer entered the sample, as farmer number 1 for that location. If the farmer did not grow maize in the current year, then we identified the nearest neighbor to that farmer and repeated until farmer number 1 was identified for that location. Second, from farmer number 1 (for that location), five neighboring farmers were identified on the basis of spatial proximity and direction. We started with the nearest farmer in the direction due North (0°), and proceeded to the nearest farmer in a clockwise direction at 72° intervals. Once it was confirmed that they grew maize in the current season, they were added to the sample. If any of these farmers did not grow maize, their nearest neighbor (not otherwise already included in the sample) was evaluated for suitability, until a total of 6 farmers for the selected point location were identified. If, at the time of the survey, a sample farmer was not available (or unable to be enumerated due to death, leaving the village or no longer planting maize), a replacement was made following the same spatial proximity rules as used in the initial selection. This replacement farmer was given the same household identification number as the drop-out farmer. At baseline data collection, around 27 originally selected households (3.6 percent of the sample) were replaced.

Table 1 summarizes the number of grid cells and households selected in each zone. From the total of 130 1 km × 1 km grid cells, 40 of them are located in West Gojjam, 34 in East Showa and 28 each in Bako and Jimma.

**Table 1:** Summary of number of grid cells and households by zone.

Administrative zones	Number of grid cells	Number of households
West Gojjam	40	240
East Shewa	34	166
Jimma	28	165
Bako	28	167
Total number of grid cells	130	738

The sample size was chosen on the basis of power calculations carried out for our main outcome variables: the absolute difference between recommended and actual fertilizer use in kg/ha for the maize area planted; total maize production in kg/ha; profit from maize production in Ethiopian Birr per hectare; and average per-capita consumption expenditure.^7^ Randomization took place at the grid-cell level with each grid cell randomly assigned to one of three groups. We stratified by four blocks, defined by administrative zones. Since the total number of grid cells cannot be evenly assigned to the three groups, we first randomly assigned an extra grid cell to one of the three groups. The first treatment arm was randomly selected to get one extra grid cell. This means that treatment arm one has 44 grid cells, whereas treatment arm two and the control group have 43 grid cells each.

In addition, while each zone should have an equal number of grid cells across the three groups, the uneven number of grid cells prevents this (see Table 1 above). Hence, we randomly allocate the extra grid cell in each zone to a particular group. Two zones take an extra grid cell in treatment group one given that there is an extra grid cell assigned to this group, with the Bako and East Shewa zones randomly selected to have an extra grid cell. Jimma and West Gojjam are randomly selected to have an extra grid cell in treatment group two and the control group, respectively. Table 2 presents the number of grid cells and households randomly selected to treatment and comparison groups in each zone.

**Table 2:** Summary of the randomization by zone.

Group	West Gojjam	East Showa	Jimma	Bako	Total	Number of grid cells
Treatment one (T1)	78	58	54	58	248	44
Treatment two (T2)	78	54	60	53	245	43
Control (C)	84	54	53	54	245	43
Total	240	166	167	165	738	130

Households in the first treatment group received SSI, consisting of a recommended amount and blend of fertilizer to use on a particular maize plot, the optimal timing of the application of that fertilizer, and the expected yield outcome for that recommendation.^8^ The information was provided both verbally and on paper. Comparing outcomes ex-post with the control group will allow us to test whether fertilizer recommendations are more likely to be adopted when accompanied by agronomic information.

Similarly, households in the second treatment group received the same SSI as in treatment arm one and insurance cover to mitigate the risk associated with potential crop failure. For each farmer in this group, we purchased crop insurance from Oromia Insurance Company (OIC) and informed them that they were insured while we provided the site-specific recommendation. The cover included any crop failure associated with drought, flood, excess rain, fire, storms, and hail for the 2018 agricultural season. This information was explained face-to-face to farmers during the planting period. As discussed above, the aim of this treatment is to allow us to test whether the downside risk associated with fertilizer investment plays a role in the take-up of the site-specific fertilizer use recommendation.^9^ During our sample period, around 25.6 percent of farmers experienced exogenous production shocks (drought, flooding, pests and diseases). Moreover, in the 2017 agricultural season, farmers lost around 14.7 percent of their crop income due to weather-related shocks. As such, weather-related risks are salient for these farmers. Despite this, the rate of crop insurance in rural farm households in Ethiopia is very low. The baseline data shows that less than one percent of households in our sample bought crop insurance in the main maize producing areas of Ethiopia.^10^

Our primary outcomes of interest are fertilizer usage (urea, nitrogen-phosphate with sulphur (NPS), and macronutrients – nitrogen, phosphorus, and sulphur) and the adoption of fertilizer recommendations, measured as the absolute gap between the actual fertilizer usage and the recommended value. The secondary outcomes we consider are productivity, profits and household welfare. Productivity is measured as maize yield in kg/ha. Profits from maize production are measured as total revenue from maize production less the cost of fertilizer.^11^ Household welfare is measured as household per capita consumption expenditure including the values of home production, purchased commodities, and gifts.

## 4 Data and empirical approach

The baseline household and community level data^12^ were collected for 738 households at the time of harvest in 2017, from October 11 to 4 December.^13^ We collected detailed information on household composition, asset endowments, income sources, farm-level management, and the agronomic information farmers received in the past including fertilizer recommendations. We also collected plot-level data on outputs, inputs and plot management history.^14^

The interventions were carried out from March 11 to April 1 2018, just before the planting period started, for the 248 and 245 households in treatment group one and two, respectively. The follow-up survey was implemented during the harvest season of 2018, from October to December. Table 3 presents the pre-treatment descriptive statistics and balancing tests for each of the treatment arms and the control group.

**Table 3:** Mean difference between households in the treatment and control groups.

Variables	C	T1	T2	[C-T1]	[C-T2]	[T1-T2]
Fertilizer use (kg/ha)	276.1	274.1	263.2	2.01	10.89	10.89
				[0.921]	[0.445]	[0.571]
Maize production (kg/ha)	3247	3246	3205	1.41	42.27	40.86
				[0.990]	[0.726]	[0.735]
Profit from maize (Birr/ha)	17,569	18,326	19,217	−757.3	−1648*	−890.8
				[0.395]	[0.085]	[0.333]
Per-capita income	4349	4123	3989	225.8	359.7	133.9
				[0.527]	[0.301]	[0.700]
Household size	6.01	6.29	6.37	−0.28	−0.09	−0.09
				[0.175]	[0.080]	[0.677]
Number of adult members	3.40	3.36	3.48	0.04	−0.12	−0.12
				[0.763]	[0.518]	[0.351]
Household head sex	0.93	0.94	0.91	−0.00	0.02	0.02
				[0.835]	[0.394]	[0.288]
Household head age	45.67	44.60	45.02	1.07	−0.42	−0.42
				[0.337]	[0.564]	[0.705]
Maximum years of education	13.96	14.88	13.85	−0.92	1.03	1.03
				[0.685]	[0.960]	[0.654]
Fertilizer use dummy	0.96	0.97	0.98	−0.01	−0.01	−0.01
				[0.445]	[0.191]	(0.574]
Credit take-up rate	0.33	0.31	0.38	0.02	−0.07	−0.07
				[0.633]	[0.257]	[0.106]
Flood dummy	0.08	0.05	0.06	0.03	−0.00	−0.00
				[0.195]	[0.287]	[0.818]
Drought dummy	0.05	0.04	0.04	0.01	−0.00	−0.00
				[0.486]	[0.663]	[0.795]

Note: All data are from the 2017 APS data set. Columns 1 to 3 present the summary statistics for households in the comparison (C), treatment one (T1) and treatment two (T2) groups, respectively. Columns 4 to 6 show the mean difference between control group and treatment arm one [C-T1], the control group and treatment two [CT2], and treatment arm one and treatment arm two [T1-T2], respectively. P-values are reported in brackets.

Column 1 presents the summary statistics for households in the control group (C), and columns 2 and 3 present the summary statistics for households in treatment groups one (T1) and two (T2), respectively.^15^ Columns 3 to 6 show the mean difference between households in the control and treatment arm one (C-T1), control and treatment arm two (C-T2), and treatment arms one and two (T1-T2), respectively. The groups are well balanced across all of the main characteristics with no significant differences in means detected.^16^ We also examine if there are significant differences in means between treatment and control groups within each zone and find that within zones our data are also well balanced.^17^

We asked farmers at baseline whether they were aware of the current regional fertilizer recommendations given by the district-level agricultural development agents, and whether they followed the recommendation. In the baseline survey, around 65 percent of farmers knew these regional fertilizer recommendations. Among those who knew the recommendation, 65 percent reported that they followed the recommendation, although only around one-third of these actually applied close to the recommended amount.^18^

Table 4 presents the summary statistics on the actual average baseline fertilizer application rates for farmers in each group relative to the coarse (zonal-level) fertilizer recommendation rates. Instead of using the total amount of inorganic fertilizer in kg/ha, we focus on the level of macronutrients applied and the recommended amount. As revealed in the first panel of the table, farmers in treatment arm one (SSI only) used about 152.3 kg/ha of nitrogen, phosphorus and sulphur for maize production at baseline. This figure is lower than the average coarse macronutrient recommended rate of 208.4 kg/ha. The data also reveal that at baseline around 77.7 percent of farmers in treatment arm one applied macronutrients below the recommended rate. Similarly, in the second panel of Table 4, we see that farmers in treatment arm two (SSI coupled with insurance) used around 146 kg/ha of nitrogen, phosphorus and sulphur for maize production at baseline, lower than the blanket recommendation rate. On average, around 78.4 percent of farmers in treatment arm two applied a macronutrient rate below the site-specific recommended rate. Similarly, in the third panel of Table 4, we find that around 74.1 percent of farmers in the control group apply fertilizer at or below the recommended rate. The differences across groups are not statistically significant.

**Table 4:** Descriptive statistics and balance tests for actual and recommended fertilizer application rates at baseline.

	N (kg/ha)	P_2_O_5_ (kg/ha)	S (kg/ha)	All (kg/ha)
*Panel A: Treatment one (T1)*				
Farmers baseline actual nutrient application rates	90.3	52.4	9.7	152.3
	(83.6)	(48.9)	(9.0)	(136.7)
Coarse (zonal level) recommended value	122.6	71.8	14	208.4
	(13.8)	(7.9)	(0.0)	(21.7)
Nutrient gap	32.3	19.1	4.3	55.6
	(77.8)	(46.5)	(9.0)	(128.3)
Share of farmers below the recommended rate (%)	74.8	76.0	76.5	77.7
*Panel B: Treatment two (T2)*				
Baseline nutrient application rates	84.7	52.2	9.6	146
	(60.3)	(32.9)	(6.0)	(94.3)
Coarse (zonal level) recommended value	122.3	72.0	14	209
	(13.5)	(7.8)	(0.0)	(21.3)
Nutrient gap	38.6	19.9	4.4	62.9
	(53.6)	(30.4)	(6.0)	(84.3)
Share of farmers below the recommended rate (%)	75.8	74.1	75.0	78.4
*Panel C: Control (C)*				
Baseline nutrient application rates	88.2	53.6	9.9	152
	(68.0)	(37.7)	(7.0)	(107.3)
Coarse (zonal level) recommended value	122.8	71.8	14	208.7
	(13.6)	(7.9)	(0.0)	(21.5)
Nutrient gap	35.0	18.2	4.1	57.0
	(61.5)	(34.9)	(7.0)	(97.4)
Share of farmers below the recommended rate (%)	73.2	71.5	72.0	74.1
*Panel D: Differences in means (C-T1)*				
Baseline nutrient application rates	−1.92	0.88	0.16	−0.87
	[0.78]	[0.83]	[0.83]	[0.94]
Coarse (zonal level) recommended value	0.28	0.16	0.00	0.44
	[0.83]	[0.83]	[1.00]	[0.83]
Nutrient gap	2.20	−0.72	−0.16	1.31
	[0.73]	[0.85]	[0.83]	[0.90]
Share of farmers below the recommended rate (%)	−0.02	−0.04	−0.05	−0.04
	[0.68]	[0.30]	[0.25]	[0.34]
*Panel E: Differences in means (C-T2)*				
Baseline nutrient application rates	3.72	1.38	0.25	5.35
	[0.53]	[0.67]	[0.67]	[0.57]
Coarse (zonal level) recommended value	−0.19	−0.11	0.00	−0.30
	[0.88]	[0.88]	[1.00]	[0.88]
Nutrient gap	−3.91	−1.49	−0.25	−5.65
	[0.46]	[0.62]	[0.67]	[0.50]
Share of farmers below the recommended rate (%)	−0.03	−0.02	−0.03	−0.04
	[0.51]	[0.59]	[0.46]	[0.27]
*Panel F: Differences in means (T1-T2)*				
Baseline nutrient application rates	5.64	0.49	0.09	6.22
	[0.40]	[0.90]	[0.90]	[0.56]
Coarse (zonal level) recommended value	−0.47	−0.27	0.00	−0.74
	[0.71]	[0.71]	[1.00]	[0.71]
Nutrient gap	−6.10	−0.76	−0.09	−6.96
	[0.32]	[0.83]	[0.90]	[0.49]
Share of farmers below the recommended rate (%)	−0.01	0.02	0.02	−0.01
	[0.81]	[0.62]	[0.69]	[0.88]

Note: The macronutrients are based on the fertilizer blends used by farmers, which include urea (46% N) and NPS (19% N, 38% P and 7% S). Standard deviations are presented in parenthesis in Panels A to C. P-values of the test for the statistical significance of the differences in means between groups are reported in brackets in Panels D to F.

The identification strategy relies on randomization across grid cells. We compare outcomes at end-line between the treatment and control groups: farmers in those grid cells that are given SSI, T1; farmers in those grid-cells that are given SSI with insurance, T2; and farmers in those grid-cells that are not given any SSI or insurance, the control group. While the randomization will allow us to detect the causal impact of the interventions on fertilizer adoption, to allow for possible unobserved differences between treatment and control groups we also control for baseline values of the outcome variables of interest along with baseline characteristics. The main specification is given in equation (10).

(10) $$Y_{ib}=\beta_{0}+\beta_{1}T1_{ib}+\beta_{2}T2_{ib}+\delta_{1}Y_{ibt-1}+\delta_{2}X_{ibt-1}+\alpha_{b}+e_{ib}$$

Where $y_{ib}$ is the outcome variable for household i in block b at end-line; $T1_{ib}$ is a dummy indicator which takes a value of one if household *i* is in treatment arm one; $T2_{ib}$ is a dummy indicator which takes a value of one if household *i* is in treatment arm two; $Y_{ibt-1}$ is the value of the outcome variable at baseline; $X_{ibt-1}$ are household-specific control variables at baseline; $\alpha_{b}$ are block-specific fixed effects that will capture any differences between the four administrative zones; and $e_{ib}$ is a statistical noise term.

The coefficients $\beta_{1}$ and $\beta_{2}$ determine the causal impact of site-specific agronomic information alone and accompanied by insurance, respectively, on the outcome variable of interest. Standard errors are clustered at the village level.

We use the same specification to test for impacts on the secondary outcomes namely, productivity, profits from maize production and household welfare measured as consumption per capita.

## 5 Results

Our first outcome of interest is the total level of inorganic fertilizer used in kg/ha. The results for this outcome based on the specification presented in equation (10) are presented in Table 5.^19^ Columns 1 and 2 show the effect of the program on the average amount of inorganic fertilizer (urea and/or NPS) in kg/ha used for maize production by the household, whereas columns 3 and 4 show the effect on the amount of all macronutrients combined (nitrogen, phosphorus, and sulphur) used in kg/ha. In all estimations, we control for baseline outcome variables and block (zone) fixed effects.

**Table 5:** Impact of interventions on fertilizer application rates.

	Log fertilizer (Urea + NPS) (kg/ha)		Log all nutrients (kg/ha)	
	(1)	(2)	(3)	(4)
Treatment one	0.15* (0.09)	0.17* (0.09)	0.10 (0.08)	0.11 (0.08)
Treatment two	0.03 (0.10)	0.03 (0.10)	0.00 (0.09)	−0.00 (0.09)
Baseline outcome variables	Yes	Yes	Yes	Yes
Baseline household controls	No	Yes	No	Yes
Block/zone fixed effects	Yes	Yes	Yes	Yes
Observations	718	717	712	711

Note: The dependent variable in columns 1 and 2 is the log household level average use of total inorganic fertilizer (urea and NPS) in kilogram per hectare used for maize production at the end-line. The dependent variable in columns 3 and 4 is the log of all macronutrients combined (nitrogen, phosphorus, and sulphur) in kg/ha used at the end-line. In columns 2 and 4, we control for the outcome variables at baseline and household-specific control variables at baseline, namely, gender, education, age and marital status of the household head, household size, number of adult household members, a dummy variable for whether or not the farmer uses fertilizer, indicator for whether the household suffered from a flood or a drought, and block (zone) specific fixed effects. Kebele (village) level clustered standard errors are reported in parenthesis, with significance denoted as * p < 0.1, **p < 0.05 and ***p < 0.01.

Results show that the site-specific fertilizer recommendations increase the level of inorganic fertilizer use for maize production. The first row in column 1 shows that SSI increases the amount of fertilizer used on average for households for maize production (in kg/ha) by around 15 percent. Column 2 shows that this effect increases slightly to 17 percent when we control for household-specific control variables at baseline.^20^ When considering all nutrients combined in columns 3 and 4, however, we do not find an effect of the provision of SSI. For the treatment group that couples the provision of SSI with insurance we do not find any effect on fertilizer application for either measure.

The null effects in columns 3 and 4 for treatment arm one and in all specifications for treatment arm two could be due to heterogeneity in the actual and the recommended fertilizer application rates at the baseline. That is, farmers who applied inorganic fertilizer above the site-specific recommendation amount could reduce the amount of inorganic fertilizer used at the end-line, while those who used lower amounts of fertilizer relative to the recommended amount could increase their use. Pooling these impacts could lead to no detectable effect on average, or where there is an effect, i.e. for treatment arm one in columns 1 and 2, these opposing forces could dampen the coefficient estimates. To explore this possibility, we estimate a quantile regression model, focusing on nitrogen application rates.^21^ The results are presented in Table 6. Estimates in columns 1 and 2 show the effect of the program for the lower 10th and 30th percentiles of the distribution of the outcome variable (log N (kg/ha)), while columns 3, 4, and 5 show the results for the 50th, 70th, and 99th percentiles of the distribution. We find that for the lowest 10th percentile of the distribution of log nitrogen use, farmers in treatment arm one increased their nitrogen application rate by 19 percent at end-line. This effect decreased to 11 percent for farmers in treatment arm one for the 30th quantile of the distribution. Interestingly, we find a negative effect for the top 99th percentile of the distribution suggesting that farmers that were over-applying fertilizer at baseline in treatment arm one adjust their application rates downwards. We do not find any statistically significant effect of treatment arm two for any quantile of the distribution.

**Table 6:** Impact of interventions on fertilizer application rates (quantile regression results).

	Log all nutrients (kg/ha)				
	10th	30th	50th	70th	99th
	(1)	(2)	(3)	(4)	(5)
Treatment one	0.19** (0.08)	0.11* (0.06)	0.07 (0.07)	0.01 (0.05)	−0.22** (0.11)
Treatment two	0.10 (0.08)	0.06 (0.08)	0.04 (0.06)	0.01 (0.05)	−0.12 (0.11)
Observations	711	711	711	711	711

Note: The dependent variable in all columns is the household level average log all macronutrients combined (nitrogen, phosphorus, and sulphur) use for maize production in kilogram per hectare at the end-line. Estimates in column 1 and 2 show the effect of the program for the lower 10th and 30th percentile of the distribution of the outcome variable, whereas estimates in column 3, 4, and 5 present the effect of SSI on fertilizer application rate for the 50th, 70th, and 99th percentile of the distribution of the outcome variable. In all columns, we control for the outcome variables at the baseline, household-specific control variables at baseline, namely, gender, education, age and marital status of the household head, household size, number of adult household members, a dummy variable for whether or not the farmer uses fertilizer, indicator for whether the household suffered from a flood or a drought, and block (zone) specific fixed effects. Bootstrapped standard errors are reported in parenthesis, with significance denoted as * p < 0.1, **p < 0.05 and ***p < 0.01.

Next, we consider the impact of the interventions on the absolute difference between the recommended and the actual fertilizer use. The results are presented in Table 7. Columns 1 and 2 show the effect of the interventions on the household level average absolute difference between the recommended and actual use of nitrogen in kg/ha used for maize production, while columns 3 to 4 and columns 5 to 6 show the absolute gap for phosphorus and sulphur used for maize production, respectively. Columns 7 and 8 present the results for the overall average of all three nutrients. For the treatment households, the fertilizer use gap is calculated by using the absolute difference between the actual macronutrient in kg/ha that farmers applied for maize production and the site-specific recommended values, whereas for the control households we used the absolute deviation of the actual macronutrients used by farmers and the regional recommendation values. In all estimations, we control for baseline outcome variables, household-specific control variables at baseline (with the exception of column 1), and block (zone) fixed effects.

**Table 7:** Impact of interventions on absolute difference between recommended and actual fertilizer use.

Variables	N (kg/ha)		P2O5 (kg/ha)		S (kg/ha)		All nutrients (kg/ha)	
	(1)	(2)	(3)	(4)	(5)	(6)	(7)	(8)
Treatment one	−13.22*** (4.10)	−13.20*** (4.13)	−4.00* (2.35)	−4.18* (2.38)	−1.28*** (0.45)	−1.32*** (0.46)	−17.92*** (6.35)	−18.29*** (6.49)
Treatment two	−13.45*** (4.08)	−13.68*** (4.16)	−7.20*** (2.61)	−7.11*** (2.68)	−1.85*** (0.49)	−1.82*** (0.49)	−21.65*** (6.97)	−21.70*** (7.15)
Baseline control mean	88.5	88.5	53.6	53.6	9.9	9.9	151.9	151.9
F-statistic	0.00	0.01	1.43	1.19	1.30	0.99	0.28	0.23
Prob > F	(0.96)	(0.91)	(0.23)	(0.28)	(0.25)	(0.32)	(0.60)	(0.63)
Baseline outcome variable	Yes	Yes	Yes	Yes	Yes	Yes	Yes	Yes
Baseline household controls	No	Yes	No	Yes	No	Yes	No	Yes
Block (zone) fixed effect	Yes	Yes	Yes	Yes	Yes	Yes	Yes	Yes
Observations	702	701	702	701	702	701	712	711

Note: The dependent variable in columns 1 and 2 is the household level average absolute difference between recommended and actual use of nitrogen in kilograms per hectare used for maize production. In columns 3 to 4 and columns 5 to 6 the dependent variable is the household average absolute difference between recommended and actual use of phosphorus and sulphur used for maize production, respectively. Column 7 and 8 presents the results for the average absolute difference for all macronutrients combined. In columns 2, 4, 6 and 8, we control for the outcome variables at baseline, household-specific control variables at baseline, namely, gender, education, age and marital status of the household head, household size, number of adult household members, a dummy variable for whether or not the farmer uses fertilizer, indicator for whether the household suffered from a flood or a drought, and block (zone) specific fixed effects. The F-statistic tests the equality of the coefficients for treatment arms one and two. Village level clustered standard errors are reported in parentheses. *p < 0.10, **p < 0.05, ***p < 0.01.

Results show that well-targeted fertilizer recommendations improve fertilizer use for maize production in terms of better alignment with locally optimal rates. The first row in column 1 shows that site-specific information on how much fertilizer to use, when to use it and the expected outcomes reduces the absolute gap of actual and the recommended use of nitrogen by around 13.2 kg/ha or 15 percent compared to the baseline amount. Column 2 shows the same effect after we control for household-specific control variables at baseline. Similarly, in columns 3 to 6, we find a reduction in the absolute gap between the farmers' actual phosphorus and sulphur use and recommended values by around 4.2 and 1.3 kg/ha, respectively, for the specifications including all baseline controls. In column 8 we see that the provision of SSI closed the absolute gap between the actual and recommended amount of total macronutrients by around 18.3 kg/ha or 12 percent compared to the baseline amount. This supports our hypothesis that households are more likely to adopt fertilizer recommendations when accompanied by SSI.

The findings for treatment arm two, where information is coupled with insurance, are very similar. The second row in columns 1 and 2 show that the combined information-insurance treatment closed the absolute gap between the actual and recommended use of nitrogen use by around 13.7 kg/ha, or 15.5 percent. This effect is not statistically different from the effect of the information-only treatment. Similarly, the results in the second row of columns 3 to 8 show that the combined treatment reduces phosphorus, sulphur and total macronutrient values (nitrogen, phosphorus and sulphur) by 7.1, 1.8, and 21.7 kg/ha, respectively. These coefficients are also not statistically different from those for the information-only treatment. This suggests that the addition of the insurance product does not affect the impact of SSI.

During the end-line data collection, we asked farmers who received crop insurance cover on the relevance of the insurance scheme in making their production decisions. Only 35 percent of farmers found the insurance cover useful. Farmers reported that the main reasons behind the low perceived usefulness of the insurance cover included the fact that they never experienced shocks (40 percent), they did not trust that claims could be made in the event of a shock (48 percent), and that they did not understand what insurance was (22 percent). This is in line with recent literature which identifies both lack of trust that payouts would be made (Cai et al., 2009; Ceballos et al., 2019) and poor understanding of insurance mechanisms (Njue et al., 2018; Sibiko and Qaim, 2017) as key factors in the low demand for insurance by smallholder farmers. Dercon et al. (2014), Fonta et al. (2018), and Njue et al. (2018) all find, however, that awareness and training in crop insurance enhances uptake.^22^ While we cannot speak to the factors affecting the uptake of insurance, our results suggest that giving agricultural insurance for free and explaining its benefits to farmers, on average, does not impact on production decisions. This suggests that in this context either the downside risk of making agricultural investments is not important for farmers in making their production decisions or agricultural insurance is not the appropriate mechanism.^23^

### 5.1 Secondary outcomes

Overall, we find that households are more likely to adopt fertilizer recommendations when accompanied by SSI. The next question is whether these adjustments to fertilizer use lead to improved productivity and profits. In Table 8, we examine the impact of the two treatments on maize yields, measured in kg/ha, and profit from maize production. As revealed in columns 1 and 2, there is a noticeable increase in yields for plots cultivated by farmers who received the SSI, both in treatment arms one and two.^24^ We find a 0.5 MT/ha yield difference between plots cultivated by farmers in the treatment and control groups. Compared to the control group whose average maize production at the baseline was around 3.3 MT/ha, the introduction of SSI improves average maize productivity of the treatment group by around 15 percent.

**Table 8:** Impact of interventions on productivity, profit and consumption.

	Aggregate farm-level output (kg/ha)		Aggregate farm-level profit (Ethiopian Birr)		Log per-capita consumption expenditure	
	(1)	(2)	(3)	(4)	(5)	(6)
Treatment one	437.4*** (162.2)	457.8*** (164.8)	3541*** (1167)	3643*** (1181)	0.04 (0.06)	0.04 (0.06)
Treatment two	430.0** (174.5)	476.3*** (174.2)	2895** (1256)	3359*** (1251)	−0.04 (0.06)	−0.03 (0.06)
Baseline control mean	3247	3247	26,662	26,662	16,857	16,857
F-statistic	0.00	0.01	0.27	0.05	1.22	1.16
Prob > F	(0.97)	(0.91)	(0.61)	(0.81)	(0.27)	(0.28)
Baseline outcome variable	Yes	Yes	Yes	Yes	No	No
Baseline household controls	No	Yes	No	Yes	No	Yes
Baseline plot controls	No	Yes	No	Yes	No	No
Block (zone) fixed effect	Yes	Yes	Yes	Yes	Yes	Yes
Observations	896	892	896	892	705	704

Note: The dependent variable in columns 1 and 2 is the aggregate farm-level average maize production in kg/ha. The dependent variable in columns 3 and 4 is the average farm-level profit in Ethiopian Birr. In columns 5 and 6 the dependent variable is average per-capita consumption in Ethiopian Birr. The unit of analysis in columns 1, 2, 4, and 5 is the plot while in columns 6, and 7 it is the household, which explains the difference in the number of observations. Household-specific control variables at baseline are gender, education, age and marital status of the household head, household size, number of adult household members, a dummy variable for whether or not the farmer uses fertilizer, and indicator for whether the household suffered from a flood or a drought. Plot specific control variables are indicators for pesticide, herbicide and manure use, and indicators for intercropping and use of improved seed. The F-statistic tests the equality of the coefficients for treatment arms one and two. Village level clustered standard errors are reported in parentheses. *p < 0.10, **p < 0.05, ***p < 0.01.

To provide some reassurance that the increase in productivity is related to improved application of fertilizer we also examine whether there is a difference in the number of labour hours allocated for maize production by farmers in treatment and control groups. It is possible, for example, that the information about the appropriate level of fertilizer and the expected yields motivates farmers to worker harder thus leading to higher productivity. The results are presented in Table D1 of Appendix D in the Online Appendix and show that there is no statistically significant difference between treatment and control farmers in the total number of labour hours allocated for maize production.^25^

In columns 3 and 4, we test whether the increase in productivity translates into higher profits. We find that site-specific agronomic information significantly improves plot-level profitability (in a range of around 12.6 to 13.7 percentage points). The effects are similar for both treatment arms. This suggests that the increased fertilizer use as a result of the SSI not only improves the productivity of plots but also profits from maize production.

In Table 8 we also consider whether the increase in productivity/profits has knock-on effects for household welfare by exploring the impact of the treatments on the per-capita consumption expenditure of households. The results are presented in columns 5 and 6 and show that there is no significant difference in the average per capita consumption expenditure between treatment and control households at end-line suggesting that productivity/profit increases have not yet translated into welfare improvements. This could be due to the short time span between the harvest period and the end-line data collection.

### 5.2 Internal validity checks

Households in the comparison group receive information about the coarse/regional fertilizer recommendations from the district-level agricultural development agents. However, from the baseline data, only around 65 percent of farmers knew these regional fertilizer recommendations. It is possible therefore, that our intervention worked because it provided information to farmers on the amount of fertilizer to use and not because of the site-specific nature of the information. To separate out the impact of the site-specific recommendations from the provision of information more generally we conduct three robustness checks. First, we restrict our sample to households who were aware of the baseline recommendation and estimate equation (10). The results are presented in Panel A of Table 9 and show that the effect of the two treatments on the absolute gap between the actual and recommended fertilizer use for the restricted sample are very similar to those found for the sample as a whole. In fact, the magnitude of the coefficients are even greater for the restricted sample suggesting that the site-specific nature of the information is the important aspect of the treatment.

**Table 9:** Robustness checks.

	N (kg/ha)	P2O5 (kg/ha)	S (kg/ha)	All nutrients (kg/ha)
*Panel A: Restricted Sample*	(1)	(2)	(3)	(4)
Treatment one	−15.17*** (5.20)	−6.69** (2.90)	−1.74*** (0.55)	−22.93*** (8.34)
Treatment two	−13.50*** (5.14)	−8.93*** (3.05)	−2.11*** (0.56)	−25.44*** (8.44)
Baseline control mean	86.9	53.2	9.8	149.9
F-statistic	0.09	0.51	0.42	0.08
Prob > F	(0.77)	(0.48)	(0.52)	(0.78)
Observations	459	459	459	463
*Panel B: Heterogeneity in baseline gap*	(1)	(2)	(3)	(4)
Treatment one	−21.44*** (5.70)	−13.64*** (3.71)	−3.23*** (0.70)	−37.27*** (9.56)
Treatment two	−20.60*** (6.77)	−12.27*** (3.90)	−2.94*** (0.73)	−33.39*** (11.21)
Treatment one x Baseline gap	0.11** (0.06)	0.12*** (0.04)	0.03*** (0.01)	0.25*** (0.09)
Treatment two x Baseline gap	0.09 (0.08)	0.07 (0.04)	0.01* (0.01)	0.15 (0.13)
Baseline gap	−0.05 (0.05)	−0.07** (0.03)	−0.01** (0.01)	−0.14* (0.08)
Baseline control mean	88.5	53.6	9.9	151.3
F-statistic	0.02	0.12	0.16	0.14
Prob > F	(0.89)	(0.73)	(0.69)	(0.71)
Observations	701	701	701	711
*Panel C: Heterogeneity in baseline access to extension services*	(1)	(2)	(3)	(4)
Treatment one	−13.10** (5.76)	−4.51 (3.39)	−1.37** (0.65)	−19.43** (9.01)
Treatment two	−15.24** (5.94)	−6.95* (3.80)	−1.78** (0.71)	−20.99** (9.89)
Treatment one x Extension service	−1.80 (8.48)	−0.14 (5.10)	−0.03 (0.98)	0.54 (13.16)
Treatment two x Extension service	3.07 (8.72)	−0.56 (4.99)	−0.12 (0.95)	−2.09 (13.78)
Extension service	−5.45 (5.51)	−3.73 (3.21)	−0.57 (0.64)	−9.62 (9.07)
Baseline control mean	88.5	53.6	9.9	151.3
F-statistic	0.15	0.50	0.39	0.03
Prob > F	(0.70)	(0.48)	(0.53)	(0.86)
Observations	701	701	701	711

Note: The dependent variables in columns 1, 2 and 3 are the household level average absolute difference between recommended and actual use of nitrogen, phosphorus and sulphur, respectively, in kilograms per hectare for maize production. The dependent variable in column 4 is the average absolute difference for all macronutrients combined. In all specifications, we control for the outcome variables at the baseline, household-specific control variables at baseline, namely, gender, education, age and marital status of the household head, household size, number of adult household members, a dummy variable for whether or not the farmer uses fertilizer, indicator for whether the household suffered from a flood or a drought, and block (zone) specific fixed effects. The F-statistic tests the equality of the coefficients for treatment arms one and two. Village level clustered standard errors are reported in parentheses. *p < 0.10, **p < 0.05, ***p < 0.01.

Second, we re-estimate our original specification and include a control for the baseline absolute gap between the actual and regional/coarse recommended value. To net out the impact of the treatment on fertilizer information more generally, we also include an interaction term between the baseline gap and each of the treatment indicators. If it is general information rather than the site-specific information driving the result, we would expect most of the effect to be picked up by the interaction term. The results are presented in Panel B of Table 9 and show that the interaction term with the treatment indicators are positive, and only statistically significant for treatment one. This suggests that the impact of the treatment is greater for those whose fertilizer use was closer to the coarse/regional recommendations at baseline. This provides further evidence that it is the site-specific nature of the fertilizer use that matters.

Finally, since regional recommendations are given by the district-level agricultural extension agents, interacting the treatment with access to agricultural extension agents at baseline will capture the extent to which the treatment depends on how well-informed farmers are at baseline. The results are presented in Panel C of Table 9 and show that differential access to baseline agricultural extension service does not matter for the impact of the treatments on the absolute gap between the actual and recommended use of fertilizer at end-line.

## 6 Conclusion

Adoption of modern agricultural practices, including increased use of improved inputs, are crucial for enabling agricultural productivity growth and structural transformation. However, the existing literature shows that adoption rates for new agricultural technologies in developing countries have remained persistently low, particularly among smallholder farmers. This could be due to a variety of different constraints including liquidity constraints, information failure and risk. In this paper, we examine whether the relevance of the technology to local conditions is a constraining factor. The context for our study is Ethiopia where blanket fertilizer recommendations are used to try to encourage farmers adopt particular blends of fertilizer. These recommendations, however, may not be optimal (or even suitable) for particular soil types or agro-climatic conditions within broad areas receiving such recommendations. Using a two-level cluster randomized control trial, we test whether providing smallholder farmers with targeted, site-specific information on the specific blend of fertilizer to use on their maize growing plots makes them more likely to adjust their fertilizer use in line with the recommendation. We also test whether coupling this information with a free insurance product that protects farmers against crop failure due to weather-related events impacts on adoption rates.

Our results show that site-specific fertilizer recommendations improve fertilizer usage and this in turn has meaningful effects on the productivity of maize production and profits. Our findings suggest that poorly defined extension information may constitute an important constraint to technology adoption. As such, our work suggests that one of the ways in which the ongoing digital transformation of agriculture in developing countries may impact growth is through better alignment of agronomic recommendations with localized production contexts. This is certainly not limited to fertilizer recommendations: many other types of agronomic management information may also be enhanced by better spatial targeting. More empirical work will also help to better understand how the uptake of information from improved and better-targeted advisory services may be conditioned by complementary interventions, such as insurance and credit. While we did not find evidence that insurance affected information uptake on average in our study context, it is possible that the combined provision of improved advisory services with insurance may have important complementary effects in other contexts.

In the current era of Information and Communications Technology (ICT)-enabled innovations in the provision of advisory and other services to smallholder farmers, there exist many possible modes of presenting and bundling such services, and additional experimental research will help to further clarify the opportunities with the greatest potential impacts on smallholder production and welfare outcomes in particular settings.

## Author statement

Hailemariam Ayalew: Conceptualization, Methodology, Fieldwork, Data Curation, Writing, Reviewing, Editing; Jordan Chamberlin: Conceptualization, Methodology, Supervision, Data Curation, Writing, Reviewing, Editing; Carol Newman: Conceptualization, Methodology, Supervision, Data Curation, Writing, Reviewing, Editing.

## Data Availability

I have attached the data and code to replicate the main findings of the study.

## Footnotes

1 Christiaensen et al. (2011) found that agricultural growth is up to 3.2 times better at reducing poverty at the one dollar a day level than growth in non-agricultural sectors.

2 We use the term *adoption* to mean the adoption of site-specific fertilizer recommendation levels.

3 Agriculture is the backbone of the Ethiopian economy and is the main source of livelihoods for rural households which accounts for around 83 percent of the total population (Davis et al., 2010).

4 Further details on the Ethiopian context and the blanket recommendations are provided in Appendix A of the Online Appendix.

5 It closes the absolute gap for nitrogen, phosphorus, and sulphur by 13.2, 4.3 and 1.4 kg/ha, respectively.

6 The key argument is that better information leads to lower outcome uncertainty and hence higher investments, *ceteris paribus*.

7 Detailed power calculations are provied in Appendix B of the Supplementary Material.

8 The recommendations were defined on the basis of nutrient omissions trials carried out in the study region within the previous season. These trials are designed to empirically measure yield response to different fertilizer applications given soil nutrient status and other soil characteristics. For example, using the data generated from on-farm nutrient omission trials conducted between 2015 and 2017, Rurinda et al. (2020) generate recommendations with a nutrient expert tool in Ethiopia, Nigeria and Tanzania. The site-specific recommendations generated via the nutrient expert tool were evaluated relative to the blanket fertilizer recommendations, the soil-test based recommendations, and a control with no fertilizer applied (Rurinda et al., 2020). See Pampolino et al. (2012) and Xu et al. (2016) for more detail on the calibration and validation methods used for the Nutrient Expert tool.

9 Since we offer agronomic information together with actual insurance cover to mitigate downside risk, our experiment is different from previous studies which investigate the impact of reducing downside risk on technology adoption of modern agricultural practices. For example, instead of providing actual insurance, Emerick et al. (2016) examine the impact of reducing downside risk on fertilizer use by introducing a new flood tolerant rice variety.

10 This is likely due to a combination of demand and supply side factors including lack of awareness of farmers and insurance products that require large amounts of collateral that most farmers cannot afford.

11 We do not find any difference in the use of labour, pesticides, or herbicides across the treatment and control groups, and so we only consider fertilizer costs in the calculation of profit. The cost of fertilizer is computed using the average price of urea and NPS. Prices in 2018 were 12.34 and 13.90 Ethiopian Birr per kilogram, respectively, and in 2017, 11.5 and 14.11 Ethiopian Birr per kilogram, respectively.

12 Our data collection was part of the Agronomic Panel Survey (APS) which we collected in collaboration with CIMMYT. It is a multi-component farm household survey designed to collect detailed and spatially explicit agronomic management data from maize farmers. It also contains information on geographic, household and plot-level contextual conditions which are relevant to understand smallholder farm decisions and outcomes at the plot and household level.

13 We used Open Data Kit (ODK) to collect plot, household and community level data. Since we used computer-assisted personal interviewing for internal validation control and well-experienced enumerators, our baseline data have very few observations with missing values. In the first week of October, we provided training to 20 enumerators and district level agricultural development agents on how to use ODK to collect the data. Since the survey covers four different zones that have different harvesting periods, we started collecting household, plot and community level data in the East Showa zone where harvesting begins earlier than in the other zones. Then we proceeded to Jimma, Bako and West Gojjam in line with the timing of the harvesting period in each zone.

14 This includes information on weeding, the application of organic fertilizer, the number of years that the plot was cultivated, whether the plot was left fallow in the last agricultural season, irrigation practices, etc.

15 We do not have data on consumption per capita at baseline. Our results are robust to the inclusion of baseline income per capita as a proxy but we do not include this in our main specification due to some missing values on income.

16 Farms in treatment arm two have slightly lower profits from maize at baseline compared with farms in the control group. This is only marginally statistically significant and works against us finding an effect.

17 These tables are presented in Tables C1 to C4 of Appendix C of the Supplementary Material.

18 Farmers were first asked whether they were aware of the current government recommendations for the area. If they responded yes, then they were asked to report the amount of NPS and Urea that were recommended. They were then asked whether they followed that recommendation.

19 Differences in the number of observations across specifications are due to missing values on some of the control variables. All of the results hold when restricting the sample to the most restrictive number of observations for all specifications.

20 These include gender, education, age and marital status of the household head, household size, type of maize seed planted, number of adult household members, and credit take-up rate.

21 Due to the availability of different fertilizer types with different nutrient composition, we use amounts of nutrient use in kg/ha as an outcome variable rather than total fertilizer use in kg/ha to better capture adherence to our specific recommendations.

22 It should be noted that other studies, for example, Takahashi et al. (2016), find that improving knowledge about agricultural insurance products does not affect uptake.

23 It is also possible that farmers did not understand the benefits of the insurance product. We made every effort to minimise this possibility. Enumerators were given in-depth training on how to explain the insurance to participants. In addition to an oral explanation participants were also given a piece of paper which stated that they would be reimbursed if they lose their crop due to drought, fire, storm or flooding.

24 We do not find any differential effect of the information coupled with the insurance treatment.

25 We also find no statistically significant difference in the effect of the treatment on the allocation of labor hours to other crops suggesting that farmers are not changing the focus of the labor efforts in response to the treatment. Results are also presented in Table D1 of Appendix D of the Online Appendix.
