# Supplementary material for: Site-specific agronomic information and technology adoption: A field experiment from Ethiopia
Source: J Dev Econ. 2022 May;156:102788. doi: 10.1016/j.jdeveco.2021.102788 (PMC8960997; doi:10.1016/j.jdeveco.2021.102788)
Supplement: Multimedia component 1 [file mmc1.docx]

**Site-Specific Agronomic Information and Technology Adoption: A Field Experiment from Ethiopia**

Online Appendix

**Appendix A: Fertilizer recommendations in Ethiopia**

Agriculture is the backbone of the Ethiopian economy and is the main source of livelihoods for rural households. More than 83 percent of the population depend directly on agriculture and many others on agriculture-related cottage industries, such as food oil processing, leather, and textiles (Davis et al., 2010). The sector also contributes 44 percent of the nation’s Gross Domestic Product (GDP), 85 percent of exports and 85 percent of employment (Spielman et al., 2010; UNDP, 2014; World Bank, 2016).

Before the 1960s, the soil in Ethiopia was rich in nutrients. This was due to sufficient per-capita arable land and a small population growth rate.^[[1]](#footnote-1)^ Consequently, farmers traditionally used a very low amount of fertilizer. As time progressed, the nation’s population grew, and the total land holding declined. For example, between 1960 and 2008, the total per-capita land area fell from 0.5 ha to 0.2 ha (Spielman et al., 2010). As a result, soil degradation and nutrient depletion have become a serious threat to agricultural productivity and food security in Ethiopia (Kebede and Yomoch, 2009). Murphy (1968) found that nitrogen (N) and phosphorus (P) were identified as being the most deficient plant nutrients in almost all Ethiopian soils. According to the Davis et al. (2010), around 5 to 7 million people in Ethiopia are chronically food insecure. Despite diverse and complex reasons for this, declining soil fertility and soil degradation are primary contributing factors.

To keep the soil nutrient balance^^[[2]](#footnote-2)^^ at sufficient levels, in the late 1960s, the Ministry of Agriculture and Natural Resources (MoANR) introduced a national-level blanket recommendation for use of urea and Diammonium phosphate (DAP) on the land to boost the N and P content (Murphy, 1968; Kassahun, 2015).^^[[3]](#footnote-3)^^ However, the application rate has remained at a low level. For example, the amount of fertilizer used in 1997 and 1999 was only 13 and 16 kg per hectare on average, respectively (FAOSTAT, 2005). Fertilizer use increased slightly to 17 and 18.5 kg per hectare in 2002 and 2015, respectively (World Bank, 2018). Spielman et al. (2010) reported that only 37 percent of farmers were using inorganic fertilizer in 2008 and the amount being applied was very low compared to other parts of the world. For instance, between 1997 and 2005 the average fertilizer use in South Asian countries was more than six times higher than the average use of fertilizer in Ethiopia. In addition to the low adoption rate, the blanket application of nutrients limited the effectiveness of its use (Tamene et al., 2017). It is therefore not surprising that farmers are reluctant to invest in fertilizer given the poor response of crops to its application in the past because of recommendations that were not specifically relevant to local conditions.

Indeed, in 2007, the Ethiopian Ministry of Agriculture (MoE) and Agricultural Research Centers developed regional fertilizer recommendation rates in Ethiopia. These recommendations have been disseminated to all farmers through district level agricultural extension workers and development agents for free. However, farmers have not adopted these blanket recommendations. For example, from our baseline survey in 2017, the average fertilizer use of farmers for maize production in all zones was below the average recommended rates for most zones at 120 kg of urea and 120 kg of NPS per hectare.^^[[4]](#footnote-4)^^ This setting provides the ideal context for testing farmers’ adoption responses to a new technology that tailors the fertilizer recommendation to each individual farmer.

Since the existing fertilizer recommendations in Ethiopia are blanket, there is uncertainty about how well the recommendation will work in particular area. This could lead to sub-optimal use of fertilizer with little impact on-farm productivity. In this study, we provide site-specific recommendations that are adjusted to local soil and climate conditions which, if adopted, should lead to a higher productivity and welfare benefit. We use the Nutrient Expert (NE) tool to generate the locally relevant recommendations (Pampolino et al., 2012; Xu et al., 2016).^[[5]](#footnote-5)^ The NE tool is a simple, quick, and easily implemented tool that helps an agricultural agent to generate up to 20 nutrient management recommendations per-day. Moreover, it will generate predictions of both yield and profit. To roll-out site-specific recommendations, android phones are required, and local agricultural extension workers require training on how to use the NE tool. The existing blanket recommendation is disseminated to farmers by local agricultural extension workers and so costs are relatively lower. As such, it is important to understand the extent of the benefits associated with the site-specific recommendations to ensure that they outweigh the cost of implementation.

**Appendix B: Power Calculations**

We conducted power calculations for our main outcome variable - the absolute deviation of the farmers’ actual application of macronutrients (nitrogen, phosphors, and sulphur) from the recommended rates in kilogram per hectare (kg/ha) – as well as other productivity and welfare outcomes of interest, i.e., farm-level maize productivity (kg/ha), profits from maize production in Ethiopian birr, and average per capita consumption expenditure in Ethiopian birr.^[[6]](#footnote-6)^.

To calculate the minimum detectable effect of the absolute deviation of farmers’ actual fertilizer application from the recommended amount, we estimate the intra-cluster correlation coefficient using the baseline data. For power of 0.8, cluster size of 6, test size of 0.05 and intra cluster correlation of 0.08, we found a minimum detectable effect of 0.238 for 130 clusters. That is, we will be able to detect a treatment induced reduction of nutrient supply gaps of at least 23.8 percent with only a 20% chance of a type II error, assuming a test size of 0.05.

Similarly, for the secondary outcome variables (productivity, profit, and household welfare), we estimate the minimum detectable effect for a power of 0.8, cluster size of 6 and cluster number of 130. For maize productivity, with an intra-cluster correlation of 0.12, we found a minimum detectable effect of 25.7, indicating that we will be able to detect a treatment induced improvement of farm productivity of at least 25.7 percent for plots managed by treatment farmers with only a 20% chance of a type II error, assuming a test size of 0.05. Similarly, for ab intra-cluster correlation of 12.1, we found a minimum detectable effect of 25.7 for profits from maize production. That is, we will be able to detect treatment induced improvement of plot-level profits from maize of at least 25.7 percent for plots managed by treatment farmers with only a 20% chance of a type II error, assuming a test size of 0.05.

Finally, using the intra-cluster correlation for per-capita consumption expenditure from the SLUEH survey data (0.09), we estimate the minimum detectable effect, for power of 0.8, cluster size of 6 and cluster number of 130, of 24.5 for per-capita consumption expenditure between farmers in the treatment and control groups. We will be able to detect treatment induced improvement of per-capita consumption expenditure of at least 24.5 percent for households in the treatment groups with only a 20% chance of a type II error, assuming a test size of 0.05.

**Appendix C: Baseline covariate balance tests at zonal level**

Table C1: Baseline mean difference between households in treatment one, two and the comparison groups in the West Gojjam zone

| Variables | C | T1 | T2 | [C-T1] | [C-T2] | [T1-T2] |
| --- | --- | --- | --- | --- | --- | --- |
| Fertilizer use (kg per hectare) | 433 | 461 | 403 | -28.3 | 30.2 | 58.43 |
|  |  |  |  | [0.481] | [0.286] | [0.143] |
| Maize production (kg per hectare) | 3391 | 3502 | 3304 | -111 | 86.8 | 197.7 |
|  |  |  |  | [0.553] | [0.619] | [0.281] |
| Profit (Ethiopian birr per hectare) | 16,358 | 16,069 | 16,938 | 288.9 | -580.6 | -869.5 |
|  |  |  |  | [0.856] | [0.745] | [0.601] |
| Per-capita income | 2804 | 3143 | 3007 | -339 | -202 | 136.8 |
|  |  |  |  | [0.515] | [0.699] | [0.793] |
| Household size | 5.64 | 5.81 | 5.86 | -0.16 | -0.22 | -0.05 |
|  |  |  |  | [0.566] | [0.517] | [0.874] |
| Number of adult members | 3.29 | 3.33 | 3.31 | -0.05 | -0.02 | 0.03 |
|  |  |  |  | [0.802] | [0.910] | [0.902] |
| Household head sex | 0.98 | 0.97 | 0.94 | 0.00 | 0.04 | 0.04 |
|  |  |  |  | [0.941] | [0.210] | [0.249] |
| Household head age | 46.37 | 45.7 | 46.28 | 0.68 | 0.09 | -0.59 |
|  |  |  |  | [0.727] | [0.964] | [0.775] |
| Marital status | 2.06 | 2.19 | 2.04 | -0.13 | 0.02 | 0.15 |
|  |  |  |  | [0.180] | [0.770] | [0.123] |
| Maximum years of education | 15.68 | 17.9 | 19.72 | -2.19 | -4.04 | -1.85 |
|  |  |  |  | [0.638] | [0.395] | [0.721] |
| Fertilizer use dummy | 1.00 | 1.00 | 1.00 | 0.00 | 0.00 | 0.00 |
|  |  |  |  | [1.00] | [1.00] | [1.00] |
| Credit take-up rate | 0.42 | 0.29 | 0.41 | 0.12 | 0.01 | -0.12 |
|  |  |  |  | [0.107] | [0.935] | [0.133] |
| Flood dummy | 0.05 | 0.03 | 0.04 | 0.02 | 0.01 | -0.01 |
|  |  |  |  | [0.462] | [0.776] | [0.652] |
| Drought dummy | 0.00 | 0.00 | 0.00 | 0.00 | 0.00 | 0.00 |
|  |  |  |  | [1.00] | [1.00] | [1.00] |

Note: Columns 1 to 3 present the summary statistics of households in the comparison (C), treatment one (T1) and two (T2), respectively. Column 4 to 6 show the mean difference between control and treatment arm one [C-T1], comparison and treatment two [CT2], and treatment arm one and two [T1-T2] groups respectively. ^*^ *p* < 0.10, ^**^ *p* < 0.05, ^***^ *p* < 0.01.

Table C2: Baseline mean difference between households in treatment one, two and the comparison groups in the East Shoa zone

| Variables | C | T1 | T2 | [C-T1] | [C-T2] | [T1-T2] |
| --- | --- | --- | --- | --- | --- | --- |
| Fertilizer use (kg per hectare) | 70.8 | 68.5 | 75.8 | 2.34 | -4.98 | -7.32 |
|  |  |  |  | [0.832] | [0.666] | [0.512] |
| Maize production (kg per hectare) | 2975 | 2590 | 2826 | 385.7 | 149.4 | -236.3 |
|  |  |  |  | [0.115] | [0.582] | [0.362] |
| Profit (Ethiopian birr per hectare) | 18,144 | 18,141 | 18,609 | 3.21 | -464.9 | -468.1 |
|  |  |  |  | [0.999] | [0.795] | [0.814] |
| Per-capita income | 6603 | 5880 | 5427 | 723.3 | 1174 | 450.8 |
|  |  |  |  | [0.416] | [0.165] | [0.601] |
| Household size | 6.19 | 6.48 | 6.57 | -0.30 | -0.39 | -0.09 |
|  |  |  |  | [0.553] | [0.381] | [0.831] |
| Number of adult members | 3.22 | 3.05 | 3.67 | 0.17 | -0.44 | -0.61** |
|  |  |  |  | [0.536] | [0.124] | [0.031] |
| Household head sex | 0.89 | 0.88 | 0.80 | 0.01 | 0.09 | 0.08 |
|  |  |  |  | [0.876] | [0.190] | [0.236] |
| Household head age | 42.8 | 41.6 | 47.7 | 1.16 | -4.96** | -6.12*** |
|  |  |  |  | [0.606] | [0.040] | [0.008] |
| Marital status | 2.46 | 2.47 | 2.70 | -0.00 | -0.24 | -0.24 |
|  |  |  |  | [0.988] | [0.236] | [0.241] |
| Maximum years of education | 7.04 | 8.14 | 14.6 | -1.10 | -7.54** | -6.44* |
|  |  |  |  | [0.547] | [0.028] | [0.082] |
| Fertilizer use dummy | 0.85 | 0.88 | 0.91 | -0.03 | -0.06 | -0.03 |
|  |  |  |  | [0.673] | [0.380] | [0.635] |
| Credit take-up rate | 0.28 | 0.31 | 0.33 | -0.03 | -0.06 | -0.02 |
|  |  |  |  | [0.709] | [0.535] | [0.797] |
| Flood dummy | 0.28 | 0.09 | 0.07 | 0.19** | 0.20** | 0.01 |
|  |  |  |  | [0.008] | [0.005] | [0.815] |
| Drought dummy | 0.11 | 0.12 | 0.11 | -0.01 | 0.00 | 0.01 |
|  |  |  |  | [0.876] | [1.000] | [0.876] |

Note: Columns 1 to 3 present the summary statistics of households in the comparison (C), treatment one (T1) and two (T2), respectively. Column 4 to 6 show the mean difference between control and treatment arm one [C-T1], comparison and treatment two [CT2], and treatment arm one and two [T1-T2] groups respectively. ^*^ *p* < 0.10, ^**^ *p* < 0.05, ^***^ *p* < 0.01.

Table C3: Baseline mean difference between households in treatment one, two and the comparison groups in the West Shoa and West Wollega zones (Bako area)

| Variables | C | T1 | T2 | [C-T1] | [C-T2] | [T1-T2] |
| --- | --- | --- | --- | --- | --- | --- |
| Fertilizer use (kg per hectare) | 341 | 315 | 325 | 25.9 | 15.37 | 15.37 |
|  |  |  |  | [0.225] | [0.503] | [0.555] |
| Maize production (kg per hectare) | 3889 | 3940 | 4067 | -51.6 | -178 | -178 |
|  |  |  |  | [0.826] | [0.475] | [0.574] |
| Profit (Ethiopian birr per hectare) | 21,717 | 20,275 | 23,348 | 1,443 | -1,631 | -3,073* |
|  |  |  |  | [0.436] | [0.447] | [0.060] |
| Per-capita income | 5830 | 5183 | 6104 | 647 | -273 | -920 |
|  |  |  |  | [0.359] | [0.709] | [0.238] |
| Household size | 5.70 | 6.24 | 6.04 | -0.54 | -0.33 | -0.33 |
|  |  |  |  | [0.221] | [0.460] | [0.630] |
| Number of adult members | 3.67 | 3.59 | 3.49 | 0.08 | 0.18 | 0.18 |
|  |  |  |  | [0.783] | [0.569] | [0.741] |
| Household head sex | 0.89 | 0.95 | 0.92 | -0.06 | -0.04 | -0.04 |
|  |  |  |  | [0.263] | [0.531] | [0.628] |
| Household head age | 47.9 | 46.8 | 42.00 | 1.04 | 5.85** | 5.85** |
|  |  |  |  | [0.669] | [0.015] | [0.039] |
| Marital status | 2.48 | 2.19 | 2.36 | 0.29 | 0.12 | 0.12 |
|  |  |  |  | [0.113] | [0.553] | [0.336] |
| Maximum years of education | 18.4 | 19.9 | 10.28 | -1.49 | 8.09* | 8.09* |
|  |  |  |  | [0.781] | [0.050] | [0.035] |
| Fertilizer use dummy | 1.00 | 1.00 | 1.00 | 0.00 | 0.00 | 0.00 |
|  |  |  |  | [1.00] | [1.00] | [1.00] |
| Credit take-up rate | 0.37 | 0.41 | 0.43 | -0.04 | -0.06 | -0.06 |
|  |  |  |  | [0.642] | [0.507] | [0.832] |
| Flood dummy | 0.02 | 0.10 | 0.08 | -0.08 | -0.06 | -0.06 |
|  |  |  |  | [0.064] | [0.166] | [0.611] |
| Drought dummy | 0.00 | 0.00 | 0.02 | 0.00 | -0.02 | -0.02 |
|  |  |  |  | [1.00] | [0.315] | [0.298] |

Note: Columns 1 to 3 present the summary statistics of households in the comparison (C), treatment one (T1) and two (T2), respectively. Column 4 to 6 show the mean difference between control and treatment arm one [C-T1], comparison and treatment two [CT2], and treatment arm one and two [T1-T2] groups respectively. ^*^ *p* < 0.10, ^**^ *p* < 0.05, ^***^ *p* < 0.01.

Table C4: Baseline mean difference between households in treatment one, two and the comparison groups in the Jimma zone

| Variables | C | T1 | T2 | [C-T1] | [C-T2] | [T1-T2] |
| --- | --- | --- | --- | --- | --- | --- |
| Fertilizer use (kg per hectare) | 170.9 | 181.1 | 195.6 | -10.1 | -24.7 | -14.5 |
|  |  |  |  | [0.678] | [0.176] | [0.555] |
| Maize production (kg per hectare) | 2652 | 2820 | 2672 | -168 | -19.5 | 148.6 |
|  |  |  |  | [0.465] | [0.935] | [0.546] |
| Profit (Ethiopian birr per hectare) | 13,839 | 17,262 | 16,828 | -3,423** | -2,989* | 434.3 |
|  |  |  |  | [0.036] | [0.062] | [0.822] |
| Per-capita income | 3108 | 2600 | 2245 | 508 | 863.2 | 355.3 |
|  |  |  |  | [0.371] | [0.068] | [0.392] |
| Household size | 6.72 | 6.81 | 7.15 | -0.10 | -0.43 | -0.34 |
|  |  |  |  | [0.828] | [0.332] | [0.474] |
| Number of adult members | 3.49 | 3.50 | 3.53 | -0.01 | -0.04 | -0.03 |
|  |  |  |  | [0.970] | [0.863] | [0.897] |
| Household head sex | 0.96 | 0.94 | 0.98 | 0.02 | -0.02 | -0.04 |
|  |  |  |  | [0.666] | [0.491] | [0.264] |
| Household head age | 45.32 | 43.91 | 43.60 | 1.41 | 1.72 | 0.31 |
|  |  |  |  | [0.555] | [0.490] | [0.898] |
| Marital status | 2.23 | 2.39 | 2.13 | -0.16 | 0.09 | 0.26** |
|  |  |  |  | [0.253] | [0.402] | [0.047] |
| Maximum years of education | 13.79 | 12.46 | 8.72 | 1.33 | 5.08 | 3.75 |
|  |  |  |  | [0.783] | [0.206] | [0.352] |
| Fertilizer use dummy | 0.96 | 1.00 | 1.00 | -0.04 | -0.04 | 0.00 |
|  |  |  |  | [0.152] | [0.131] | [1.00] |
| Credit take-up rate | 0.19 | 0.20 | 0.32 | -0.02 | -0.13 | -0.11 |
|  |  |  |  | [0.847] | [0.122] | [0.174] |
| Flood dummy | 0.00 | 0.00 | 0.05 | 0.00 | -0.05 | -0.05* |
|  |  |  |  | [1.00] | [0.101] | [0.098] |
| Drought dummy | 0.11 | 0.04 | 0.05 | 0.08 | 0.06 | -0.01 |
|  |  |  |  | [0.137] | [0.219] | [0.739] |

Note: Columns 1 to 3 present the summary statistics of households in the comparison (C), treatment one (T1) and two (T2), respectively. Column 4 to 6 show the mean difference between control and treatment arm one [C-T1], comparison and treatment two [CT2], and treatment arm one and two [T1-T2] groups respectively. ^*^ *p* < 0.10, ^**^ *p* < 0.05, ^***^ *p* < 0.01.

**Appendix D: Additional results**

Table D1: Impact of interventions on labour hours

|  | All crops  (hr./ha) | Maize production  (hr./ha) | Non-maize production (hr./ha) |
| --- | --- | --- | --- |
|  | (1) | (2) | (3) |
| Treatment one | 7.28 | 5.55 | -45.62 |
|  | (23.18) | (27.26) | (71.29) |
| Treatment two | -4.65 | -11.81 | -101.18 |
|  | (22.15) | (26.70) | (68.68) |
| Baseline controls mean | 265.5 | 328.5 | 383.8 |
| Observations | 727 | 720 | 635 |

Note: The dependent variable in column 1 is the household level average amount of labour hour per-hectare used for all crops, while in columns 2 and 3 the dependent variable is the household level average amount of labour hour per-hectare used for maize and non-maize production respectively. Household-specific control variables at baseline are gender, education, age and marital status of the household head, household size, number of adult household members, a dummy variable for whether or not the farmer uses fertilizer, indicator for whether the household suffered from a flood or a drought, and block (zone) specific fixed effects Village level clustered standard errors are reported in parentheses. ^*^ *p* < 0.10, ^**^ *p* < 0.05, ^***^ *p* < 0.01.

Table D2: Impact of interventions pesticide application

|  | All crops | Maize | Non-maize |
| --- | --- | --- | --- |
|  | (1) | (2) | (3) |
| Treatment one | -0.00 | 0.01 | 0.00 |
|  | (0.04) | (0.03) | (0.03) |
| Treatment two | -0.03 | -0.03 | 0.01 |
|  | (0.04) | (0.03) | (0.03) |
| Baseline controls mean | 0.09 | 0.03 | 0.07 |
| Observations | 727 | 727 | 680 |

Note: The dependent variable in columns 1 is a household level dummy variable for pesticides use for all crops, while in columns 2 and 3 the dependent variable is a household level dummy variable for pesticides application for maize and non-maize production respectively. Household-specific control variables at baseline are gender, education, age and marital status of the household head, household size, number of adult household members, a dummy variable for whether the farmer uses fertilizer, indicator for whether the household suffered from a flood or a drought, and block (zone) specific fixed effects. Village level clustered standard errors are reported in parentheses. ^*^ *p* < 0.10, ^**^ *p* < 0.05, ^***^ *p* < 0.01.

Table D3: Impact of intervention on herbicide application

|  | All crops | Maize | Non-maize |
| --- | --- | --- | --- |
|  | (1) | (2) | (3) |
| Treatment one | -0.06 | -0.05 | -0.03 |
|  | (0.04) | (0.04) | (0.05) |
| Treatment two | -0.01 | -0.02 | -0.02 |
|  | (0.04) | (0.05) | (0.05) |
| Baseline controls mean | 0.70 | 0.42 | 0.61 |
| Observations | 727 | 727 | 680 |

Note: The dependent variable in columns 1 is the household level dummy variable for herbicide use for all crops, while in columns 2 and 3 the dependent variable is a household level dummy variable for herbicides application for maize and non-maize production, respectively Household-specific control variables at baseline are gender, education, age and marital status of the household head, household size, number of adult household members a dummy variable for whether the farmer uses fertilizer, indicator for whether the household suffered from a flood or a drought, and block (zone) specific fixed effects. Village level clustered standard errors are reported in parentheses. ^*^ *p* < 0.10, ^**^ *p* < 0.05, ^***^ *p* < 0.01.

**References**

Ayalew, H., Admasu, Y. and Chamberlin, J., 2021. Is land certification pro-poor? Evidence from Ethiopia. Land Use Policy, 107, p.105483.

Davis, K., Swanson, B., Amudavi, D., Mekonnen, D.A., Flohrs, A., Riese, J., Lamb, C. and Zerfu, E., 2010. In-depth assessment of the public agricultural extension system of Ethiopia and recommendations for improvement. International Food Policy Research Institute (IFPRI) Discussion Paper, 1041, pp.193-201.

FAOSTAT (FAO-Statstical Database). 2005. Agriculture, Fishery, Forestry and Nutrition. CD-ROM, FAO, Rome.

Janssen, B.H., Guiking, F.C.T., van der Eijk, D., Smaling, E.M., Wolf, J. and van Reuler, H., 1990. A system for quantitative evaluation of the fertility of tropical soils (QUEFTS). Geoderma, 46(4), pp.299-318.

Kassahun, B., 2015. Soil fertility mapping and fertilizer blending. Ethiopian Agricultural Transformation Agency (Ethiopian ATA) report, Addis Ababa.

Kebede, F. and Yamoah, C., 2009. Soil fertility status and numass fertilizer recommendation of typic hapluusterts in the northern highlands of Ethiopia. World Applied Sciences Journal, 6(11), pp.1473-1480.

Makombe, G., Kelemework, D. and Aredo, D., 2007. A comparative analysis of rainfed and irrigated agricultural production in Ethiopia. Irrigation and Drainage Systems, 21(1), pp.35-44.

Murphy, H.F. 1968. A Report on Fertility Status and Other Data on Some Soils of Ethiopia. Experimental Station Bulletin No. 44. Hailesilassie College of Agriculture, Oklahoma State University.

Pampolino, M.F., Witt, C., Pasuquin, J.M., Johnston, A. and Fisher, M.J., 2012. Development approach and evaluation of the Nutrient Expert software for nutrient management in cereal crops. Computers and Electronics in Agriculture, 88, pp.103-110.

Spielman, D.J., Byerlee, D., Alemu, D. and Kelemework, D., 2010. Policies to promote cereal intensification in Ethiopia: The search for appropriate public and private roles. Food policy, 35(3), pp.185-194.

Tamene, L., Amede, T., Kihara, J., Tibebe, D. and Schulz, S., 2017. A review of soil fertility management and crop response to fertilizer application in Ethiopia: towards development of site-and context-specific fertilizer recommendation. CIAT Publication.

UNDP (United Nations Development Programme). 2014. Agricultural Growth and Transformation: Strengthening National Capacity through Sustainable Increase in Agriculture Production and Productivity. Addis Ababa, Ethiopia.

World Bank, 2016. Priorities for ending extreme poverty and promoting shared prosperity, systematic country diagnostic. Report No: 100592-ET).

World Bank. 2018. “Fertilizer Consumption (Kilogram per Hectare of Arable Land)”. https://data.worldbank.org/indicator/AG.CON.FERT.ZS

Xu, X., He, P., Pampolino, M.F., Li, Y., Liu, S., Xie, J., Hou, Y. and Zhou, W., 2016. Narrowing yield gaps and increasing nutrient use efficiencies using the Nutrient Expert system for maize in Northeast China. Field Crops Research, 194, pp.75-82.

1. From the total of land area of around 1.13 million, Ethiopia has an estimated 55 million hectares of arable land (Makombe et al., 2007). [↑](#footnote-ref-1)
2. Soil nutrient balance is the difference between nutrient inputs through, for example, organic or inorganic fertilizer, minus the nutrient loss through erosion and crop production. [↑](#footnote-ref-2)
3. A ‘blanket recommendation’ is a ‘one size fits all’ solution where all farmers use the same amount of fertilizer blend recommendation in kg per-hectare without considering crop type, soil type or agro-ecological zones. For example, it may recommend that all farmers use 100 kg Urea and 100 kg NPS per hectare for all crops in all parts of the country. [↑](#footnote-ref-3)
4. The current blanket fertilizer recommendation rate for maize production in West Gojjam, East Wollega and West Shoa are 130 kilogram nitrogen and 76 kilogram phosphate per-hectare. [↑](#footnote-ref-4)
5. Nutrient Expert is a decision-support tool (program), developed by the International Plant Nutrition Institute, that enables farmers and extension providers to quickly generate fertilizer recommendations for individual fields or for larger but similar areas, depending on the user’s requirements. It is based on the QUEFTS (Quantitative Evaluation of the Fertility of Tropical Soils) model described by Janssen et al. (1990). To make a site-specific recommendation, the tool uses information on the available fertilizer blends in Ethiopia, current farmers’ practices, relevant inputs and field history, and local conditions. Then, it provides advice on improved crop management practices, such as site-specific fertilizer blend recommendations, the potential or attainable yield a farmer can get from the same land, planting density, timing of fertilizer application, and weeding. [↑](#footnote-ref-5)
6. Since we do not have baseline data on per-capita consumption expenditure, we use the intra-cluster correlation coefficient from the Sustainable Land Use in Ethiopian Highlands (SLUEH) panel survey data. The data were collected in 2002, 2004, and 2007 in South Wollo and East Gojjam zones of the Amhara Region (Ayalew et al., 2021). [↑](#footnote-ref-6)
